# Cathepsin L and transmembrane serine protease 11E mediate trypsin-independent entry of porcine deltacoronavirus into Huh7 cells

**DOI:** 10.1128/jvi.01055-25

**Published:** 2025-08-11

**Authors:** Wenwen Xiao, Yuanxiang Xiong, Yuchen Wang, Ting Li, Chaoqun Chen, Yuting Shi, Guanning Su, Yanrong Zhou, Shaobo Xiao, Liurong Fang

**Affiliations:** 1 National Key Laboratory of Agricultural Microbiology, College of Veterinary Medicine, Huazhong Agricultural University 627716, Wuhan, China; 2 Key Laboratory of Preventive Veterinary Medicine in Hubei Province, the Cooperative Innovation Center for Sustainable Pig Production, Wuhan, China; University of Kentucky College of Medicine, Lexington, Kentucky, USA

**Keywords:** porcine deltacoronavirus (PDCoV), trypsin, cathepsin L (CTSL), transmembrane serine protease 11E (TMPRSS11E), furin

## Abstract

Porcine deltacoronavirus (PDCoV) is an emerging enteric coronavirus with zoonotic potential. Previous studies showed that PDCoV productive infection is dependent on exogenous trypsin in porcine-derived cells. In this study, we found that trypsin appears to be dispensable for PDCoV infection in human-derived cell lines. Using Huh7 cells as a model, we investigated the potential mechanisms underlying this phenomenon. Our results demonstrated that exogenous trypsin has no significant impact on PDCoV proliferation in human-derived cells. Furthermore, the culture supernatants collected from Huh7 cells can promote PDCoV infection in porcine-derived cells under trypsin-free conditions. By utilizing protease inhibitors, we identified that PDCoV enters Huh7 cells via endosomal and plasma membrane fusion pathways. Further investigations revealed that cathepsin L (CTSL) and transmembrane serine protease 11E (TMPRSS11E) mediate these pathways, respectively. Mechanistically, CTSL and TMPRSS11E cleave the PDCoV spike (S) protein, activating membrane fusion and promoting viral entry into Huh7 cells. Additionally, our results indicated that the highly expressed furin in Huh7 cells also plays a critical role in the late stages of PDCoV replication cycle, facilitating virion maturation and release. Taken together, these findings elucidate the detailed mechanisms of PDCoV infection in human-derived cells, highlighting the critical roles of CTSL and TMPRSS11E in trypsin-independent entry.

**IMPORTANCE:**

PDCoV can be isolated from human plasma samples and infect various human-derived cells, raising significant concerns regarding its potential for cross-species transmission. Coronavirus invasion involves receptor binding and spike (S) protein cleavage by proteases. While human aminopeptidase N (APN) has been confirmed as a receptor that mediates PDCoV infection, the specific proteases involved in infections of human-derived cells remain incompletely understood. Here, we investigated the mechanisms by which PDCoV enters human-derived cells and demonstrated that PDCoV infection in these cells is independent of exogenous trypsin. Furthermore, we identified two critical proteases, CTSL and TMPRSS11E, which facilitate PDCoV entry into Huh7 cells via endosomal and plasma membrane fusion pathways, respectively. Additionally, we discovered that furin promotes the maturation and release of virions. This study reveals the infection mechanisms of PDCoV in human-derived cells, highlighting the roles of CTSL, TMPRSS11E, and furin in viral entry and release.

## INTRODUCTION

Coronaviruses (CoVs) are classified into four genera based on phylogenetic analysis: *Alphacoronavirus*, *Betacoronavirus*, *Gammacoronavirus*, and *Deltacoronavirus* (1). Members of the *Alphacoronavirus* and *Betacoronavirus* primarily infect mammals including humans, members of the *Gammacoronavirus* mainly infect birds, while those of the genus *Deltacoronavirus* infect both mammals and birds (2–4). To date, porcine deltacoronavirus (PDCoV) is the sole member within the *Deltacoronavirus* genus that has been successfully isolated and cultured *in vitro* (5), making it an ideal model for investigating the pathogenesis of deltacoronaviruses. PDCoV possesses a single-stranded positive-sense RNA genome with approximately 25.4  kb, encoding 15 nonstructural proteins (nsp2-16), four structural proteins [spike (S), envelope (E), membrane (M), and nucleocapsid (N)], and three accessory proteins (NS6, NS7, and NS7a) (6). PDCoV primarily infects suckling piglets, causing severe gastrointestinal diseases characterized by diarrhea, vomiting, dehydration, and even mortality (7, 8). Since first identified in 2012 (9), PDCoV has been widespread in many countries, including the United States (10, 11), Canada (12), Korea (13, 14), Thailand (15, 16), and China (17), posing significant threats to the global swine industry.

Increasing evidence suggests that PDCoV, in addition to infecting pigs, can also infect avian species such as chickens and turkeys (18, 19), as well as other mammalian species including calves and mice (20, 21). Notably, a recent study reported the detection of PDCoV genomic RNA and successful virus isolation from plasma samples of febrile children in Haiti, suggesting the potential for PDCoV as a zoonotic pathogen (22). Consequently, some researchers have proposed that PDCoV should be considered the eighth coronavirus capable of infecting humans (23).

The entry of CoVs is typically mediated by the structural S protein, which plays a crucial role in binding to cellular receptors and facilitating virus-cell membrane fusion (24). After the binding of the viral S protein to the cell-surface receptor, the S protein undergoes cleavage, which is generally conducted by host proteases or exogenous trypsin (25). This cleavage event leads to the exposure of fusion peptide (FP) and subsequent membrane fusion between the viral envelope and the host cell membrane, thereby completing viral entry and releasing the viral genome into host cells (25, 26). For example, severe acute respiratory syndrome coronavirus 2 (SARS-CoV-2) enters cells through the interaction between S protein and angiotensin-converting enzyme 2 (ACE2) receptor (27). This interaction is followed by the cleavage of viral S protein via the host protease transmembrane serine protease 2 (TMPRSS2), which activates the membrane fusion and subsequent viral entry (25, 27). Aminopeptidase N (APN) has been identified as a receptor for PDCoV (28, 29), and APN from multiple species, including porcine, feline, chicken, and human, can mediate PDCoV infection in cell lines corresponding to species (30). Recent crystallographic studies have confirmed the binding between human APN and PDCoV S protein (31), and a CRISPR/Cas9 screen has identified APN as a critical gene involved in PDCoV infection in human Huh7 cells (32). Thus, the receptor-mediated PDCoV entry into human-derived cells has been well characterized. However, the roles of proteases during PDCoV infection in human-derived cells remain poorly understood and require further investigation.

CoVs enter host cells through two main pathways: the endosomal fusion pathway and the plasma membrane fusion pathway (33). In the endosomal fusion pathway, the virus is engulfed by endosomes, where the acidic environment activates the cleavage of the viral S protein by cathepsins, thus facilitating the fusion between the viral envelope and the endosomal membrane (34, 35). Alternatively, the viral envelope can fuse directly with the host cell plasma membrane via the plasma membrane fusion pathway (27, 36). In this pathway, the S protein is cleaved at specific sites by proteases, such as type-II transmembrane serine proteases (TTSPs), exogenous trypsin, or proprotein convertases like furin (37–40). Generally, both pathways are significant for the successful entry of CoVs into host cells, and their alternate utilization ensures efficient viral invasion across various tissues and species (35, 40). Our previous study reveals that the entry of PDCoV in porcine-derived cells predominantly relies on the trypsin-mediated plasma membrane fusion pathway (41). PDCoV has been shown to infect human-derived cells, including Huh7, A549, HeLa, HIEC-6, and HepG2 cells (30–32, 42); however, the specific proteases involved in viral entry have not been described or investigated in detail. Further research is needed to elucidate the roles of potential proteases, which will help better clarify the mechanisms underlying PDCoV infection in human-derived cells.

In this study, we demonstrated that PDCoV infection in human-derived cells does not require the supplementation of exogenous trypsin. Cysteine protease CTSL and serine protease TMPRSS11E are identified as significant proteases for PDCoV entry in Huh7 cells, mediating endosomal and plasma membrane fusion pathways, respectively. Furthermore, furin was confirmed to facilitate viral release. These findings contribute to a deeper understanding of the mechanisms underlying PDCoV infection in human-derived cells.

## RESULTS

### PDCoV infection in human-derived cells is independent of trypsin

**Fig 1:**
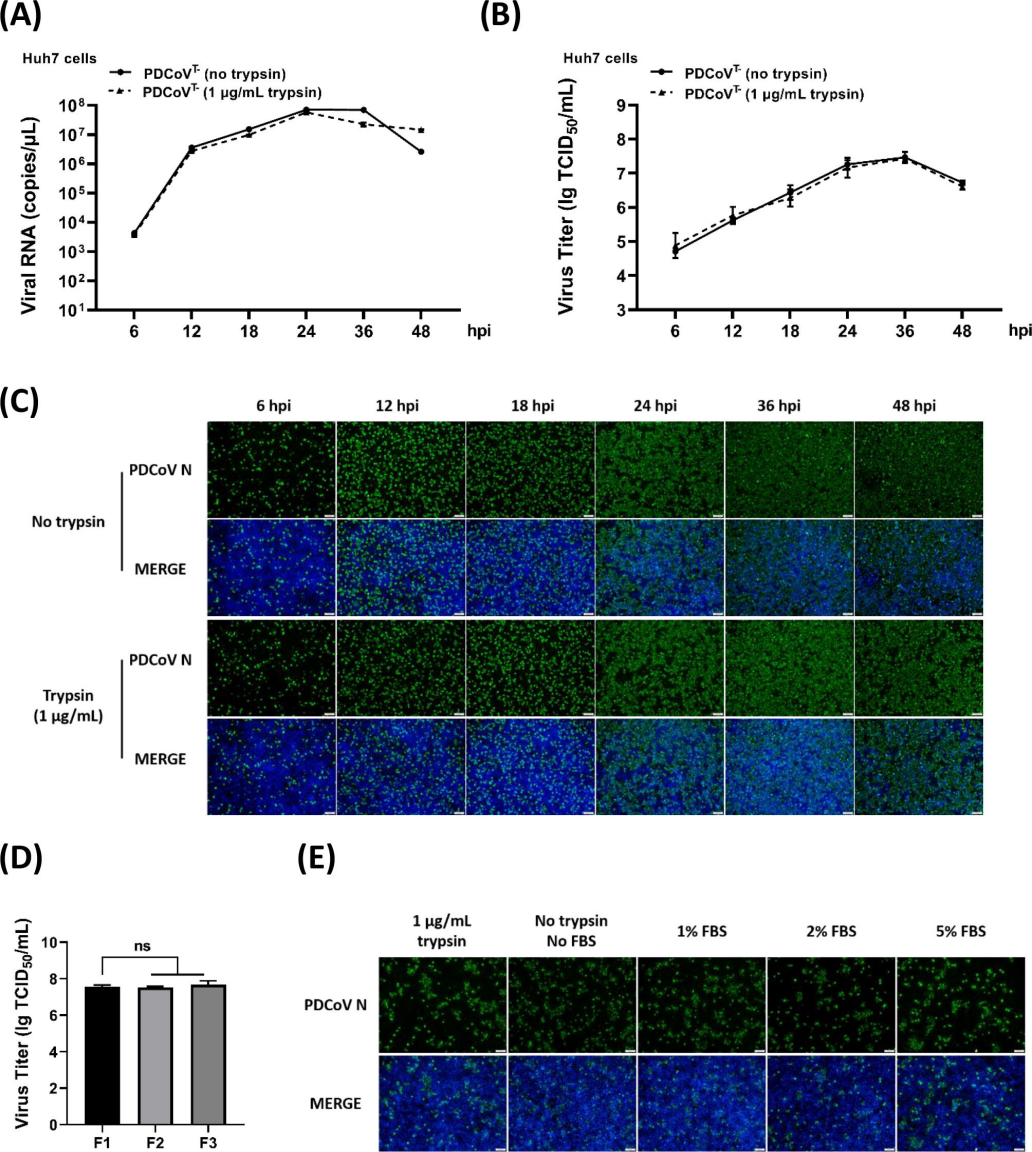
Exogenous trypsin has no significant effect on PDCoV infection in Huh7 cells. (**A–C**) One-step growth curve of PDCoV^T-^ in the presence or absence of trypsin. Huh7 cells were infected with PDCoV^T-^ with or without trypsin (1 µg/mL). At the indicated hours post-infection (hpi), viral RNA copies, titers, and N protein levels were detected by RT-qPCR (**A**), TCID_50_ (**B**), and IFA (**C**) assays, respectively. The viral N protein (green fluorescence) was stained with anti-PDCoV N antibodies, and cell nuclei (blue fluorescence) were stained with DAPI. Scale bar, 100 µm. (**D**) The viral titers of PDCoV^T-^ after successive passages on Huh7 cells. PDCoV^T-^ (MOI = 0.5) was inoculated on Huh7 cells. At 24 hpi, the cells were harvested and freeze-thawed three times, and then the viral suspensions were re-inoculated on Huh7 cells at an MOI of 0.5. The viral titer of each passage was determined by the TCID_50_ assay. (**E**) The addition of trypsin inactivator FBS. PDCoV^T-^ was inoculated on Huh7 cells with various concentrations of FBS. At the indicated hpi, IFA was performed with anti-PDCoV N antibodies. The viral N protein was stained as specific green fluorescence, and cell nuclei were stained as blue fluorescence with DAPI. Scale bar, 100 µm. ns, no significant difference, *P* > 0.05; * *P* ≤ 0.05; ** *P* ≤ 0.01; *** *P* ≤ 0.001; **** P ≤ 0.0001.

Our previous study demonstrated that trypsin is an indispensable protease for PDCoV entry and productive infection in porcine kidney epithelial (LLC-PK1) cells (41). Additionally, PDCoV has been shown to infect various human-derived cell lines, such as Huh7, HeLa, A549, and HepG2 cells (30–32, 42). To evaluate whether trypsin plays a similar role during PDCoV infection in human-derived cells, Huh7 cells were inoculated with PDCoV^T-^, which was prepared in Huh7 cells without trypsin, at a multiplicity of infection (MOI) of 0.5 in the presence or absence of trypsin. At the indicated hours post-infection (hpi), the infected cells were collected, and viral RNA copies and titers were quantified using RT-qPCR and TCID_50_ assays, respectively. As shown in Fig. 1A and B, PDCoV^T-^ infection progressively increased, reaching a peak at 36 hpi (Fig. 1A and B). Notably, there was no significant difference in viral RNA copies and titers between the trypsin-treated and untreated groups. Consistent with these findings, IFA results indicated comparable levels of PDCoV N-specific fluorescence in both trypsin-treated and untreated Huh7 cells (Fig. 1C). Similarly, the addition of trypsin had no significant effect on PDCoV^T-^ infection in other human-derived cell lines, including HeLa (Fig. S1A and B), A549 (Fig. S1C and D), and HepG2 (Fig. S1E and F) cells, indicating that PDCoV^T-^ infections in human-derived cells are independent of exogenous trypsin. Furthermore, in the absence of trypsin, viral titers were stably maintained in Huh7 cells with the increasing passages (Fig. 1D). PDCoV^T-^ infection in Huh7 cells remained unaffected even when the trypsin activity was inhibited by various concentrations of fetal bovine serum (FBS), a well-known trypsin inactivator (Fig. 1E). Collectively, these results suggest that PDCoV can establish efficient and stable infection in human-derived cells independent of trypsin. Given the higher susceptibility of Huh7 cells to PDCoV compared to other human-derived cells, Huh7 cells were selected as the model to further investigate the detailed mechanisms of PDCoV infection via a trypsin-independent pathway.

### The culture supernatants from Huh7 cells benefit PDCoV infection

**Fig 2:**
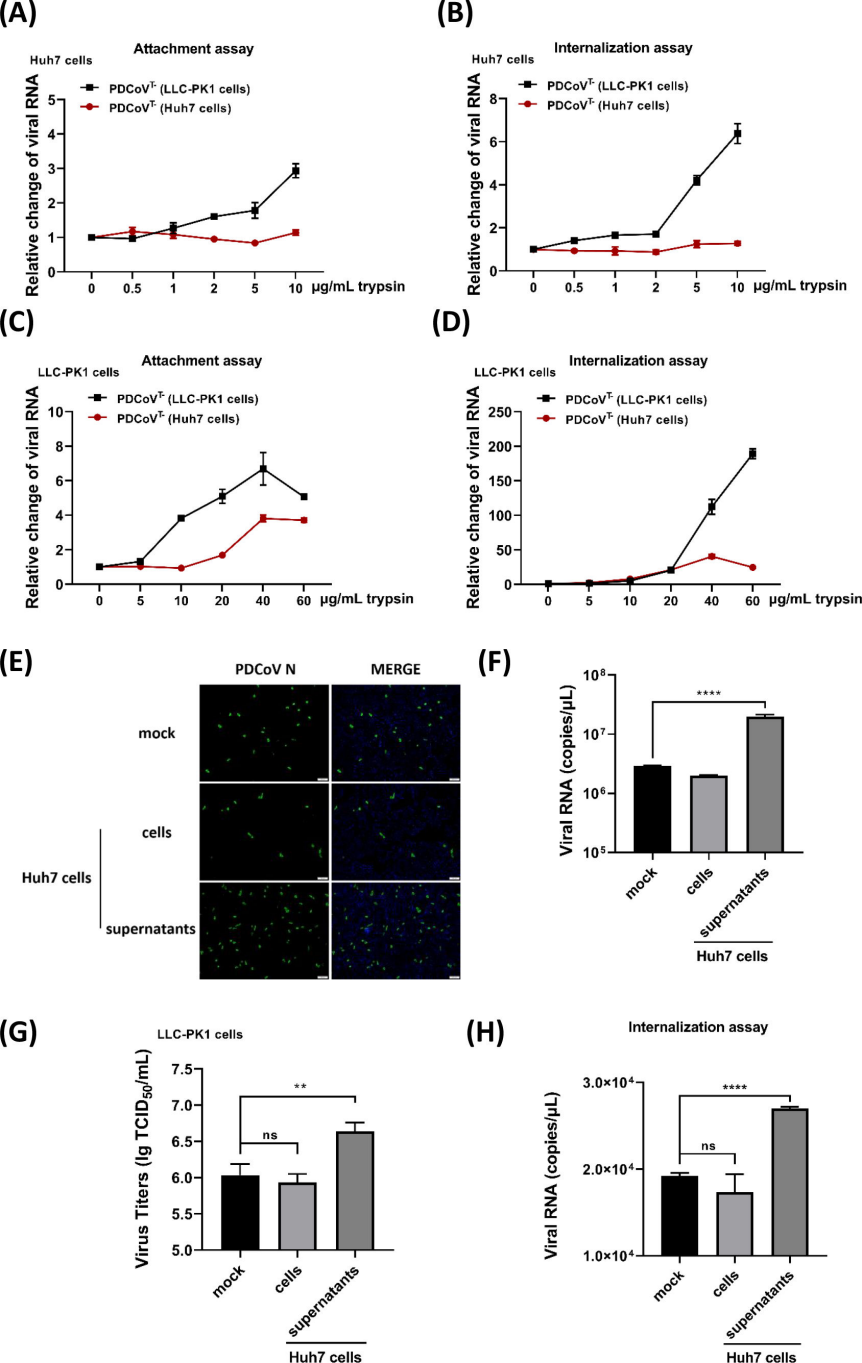
The culture supernatants of Huh7 cells benefit PDCoV infection. (**A–D**) Viral attachment and internalization assays on Huh7 (**A, B**) and LLC-PK1 (**C, D**) cells. Two types of PDCoV^T-^ virions were pretreated with trypsin at 37°C for 1 h, followed by inoculation on cells at 4℃ (for attachment) or 37℃ (for internalization) for 1 h. After the viral attachment or internalization phases, the cells were collected and washed with PBS or citric acid, respectively. The viral RNA copies of the attached and internalized viruses were measured by RT-qPCR. The viral RNA copies detected in groups without trypsin pretreatment were set as 1. (**E–H**) The supplementation of culture supernatants from Huh7 cells. Huh7 cells were cultured in DMEM with 10% FBS until reaching confluence, and then the media were replaced with basic DMEM (without FBS) for 12 h. The culture supernatants were collected, while the cells were subjected to freeze-thaw cycles with an equal volume of basic DMEM to that of the collected supernatants. The intracellular components of Huh7 cells were obtained after centrifugation and referred to as “cells.” Basic DMEM served as the untreated medium control and was referred to as “mock.” The LLC-PK1 cells were infected with PDCoV^T-^ (MOI = 0.5) with medium replacement. At 12 hpi, the viral N protein, RNA copies, and titers were detected by IFA (**E**), RT-qPCR (**F**), and TCID_50_ (**G**) assays, respectively. The viral N protein was stained as specific green fluorescence, and cell nuclei were stained as blue fluorescence with DAPI. Scale bar, 100 µm. The internalized viral RNA copies were quantified by RT-qPCR (**H**), with intracellular components of Huh7 cells and untreated culture medium serving as negative controls. ns, no significant difference, *P* > 0.05; * *P* ≤ 0.05; ** *P* ≤ 0.01; *** *P* ≤ 0.001; **** P ≤ 0.0001.

To further investigate the differences between trypsin-dependent and trypsin-independent PDCoV^T-^ infections, we propagated and purified the virions from LLC-PK1 cells and Huh7 cells, respectively. Western blot analyses revealed that the major structural proteins were specifically detected in both purified virions (Fig. S2A), and Coomassie Brilliant Blue staining clearly showed abundant PDCoV N and M proteins (Fig. S2B). Furthermore, transmission electron microscopy (TEM) demonstrated no significant morphological differences between the two types of virions, both exhibiting a characteristic crown-like structure with prominent spikes (Fig. S2C). Subsequently, both virions were inoculated into Huh7 and LLC-PK1 cells, respectively, and viral attachment and internalization assays were performed with different concentrations of trypsin. In Huh7 cells, the results showed that trypsin did not affect the attachment (Fig. 2A) and internalization (Fig. 2B) of virions derived from Huh7 cells, whereas the addition of trypsin enhanced both attachment (Fig. 2A) and internalization efficiency (Fig. 2B) of virions derived from LLC-PK1 cells. These results indicated that virions produced in different cell types exhibit discrepancies that are not easily discernible at the morphological level. Interestingly, in LLC-PK1 cells, trypsin significantly promoted the attachment (Fig. 2C) and internalization (Fig. 2D) of both virions, with a more pronounced enhancement observed during the entry of virions derived from LLC-PK1 cells. These findings suggest that the discrepancies between the two types of virions are attributable not only to subtle structural differences but also to variations in the cells' culture medium. To verify this hypothesis, culture supernatants from Huh7 cells were collected and incubated with LLC-PK1 cells. Subsequently, LLC-PK1 cells were infected with PDCoV^T-^ under trypsin-free conditions, with intracellular components of Huh7 cells and untreated culture medium serving as negative controls. At 24 hpi, IFA, RT-qPCR, and TCID_50_ assays were performed to detect the expression of PDCoV N protein, viral genome copies, and titers, respectively. The results demonstrated that higher levels of PDCoV N protein (Fig. 2E), viral RNA copies (Fig. 2F), and titers (Fig. 2G) were detected in LLC-PK1 cells whose medium was replaced with the culture supernatants of Huh7 cells, indicating the presence of unknown proteases in the supernatants that may substitute for trypsin in facilitating PDCoV^T-^ infection. Additionally, the supplementation of culture supernatants increased the internalized PDCoV^T-^ in LLC-PK1 cells (Fig. 2H), further supporting the notion that these unknown proteases promote PDCoV^T-^ entry. Collectively, these findings indicate that the PDCoV entry mechanism differs between porcine- and human-derived cells, and PDCoV employs distinct infection strategies to efficiently infect various cell types.

### PDCoV enters Huh7 cells through both endosomal and plasma membrane fusion pathways

**Fig 3:**
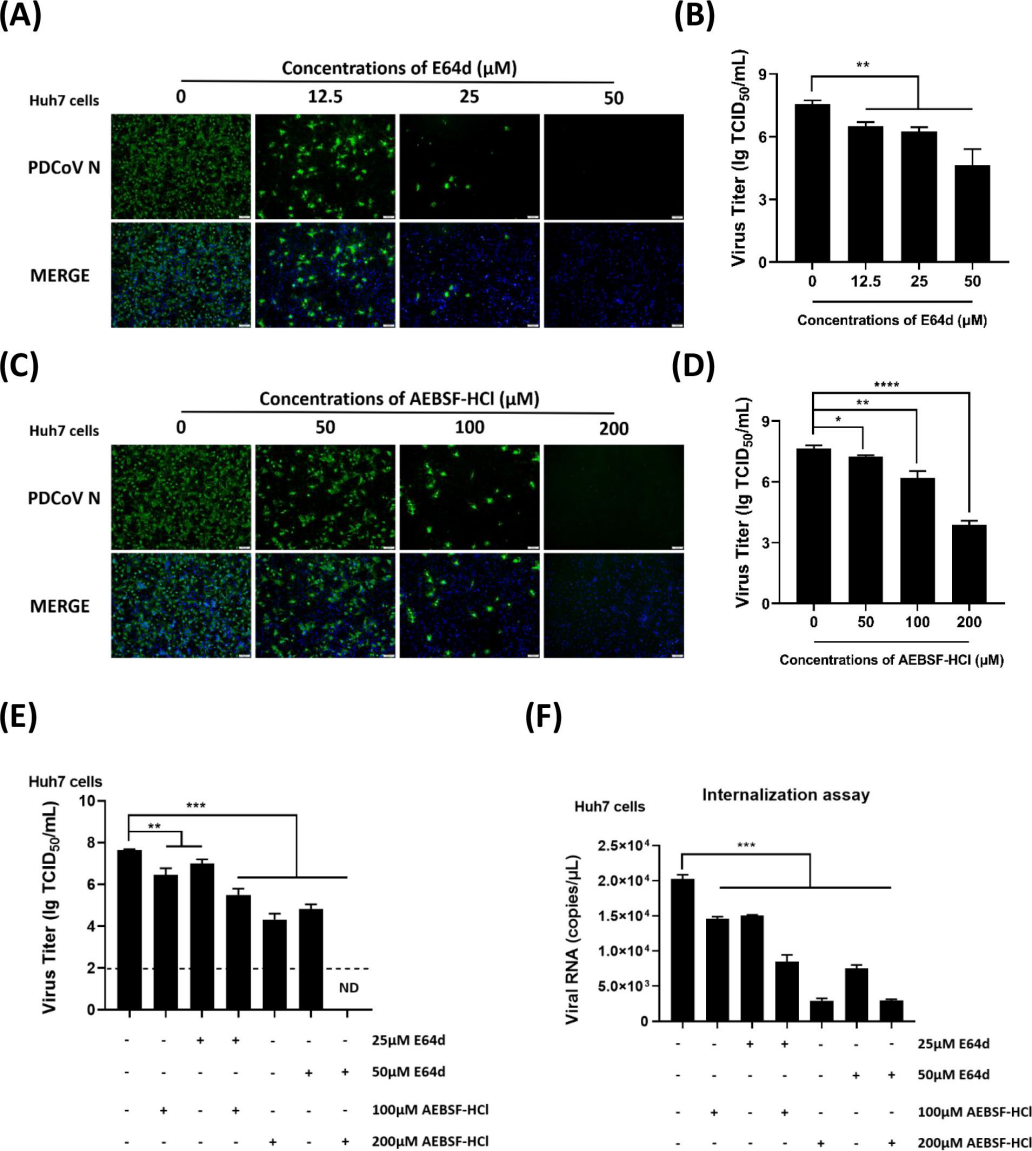
PDCoV enters Huh7 cells through both endosomal and plasma membrane fusion pathways. (**A–D**) Effects of E64d and AEBSF-HCl on PDCoV^T-^ infection. Huh7 cells were pretreated with different concentrations of E64d (0, 12.5, 25, and 50 µM) or AEBSF-HCl (0, 50, 100, and 200 µM), and then the cells were infected with PDCoV^T-^ (MOI = 0.5). At 24 hpi, the cells were collected and subjected to IFA (**A, C**) and TCID_50_ (**B, D**) assay. (**E, F**) The combined effects of E64d and AEBSF-HCl. Huh7 cells were pretreated with different concentrations of E64d (0, 25, and 50 µM) and AEBSF-HCl (0, 100, and 200 µM) concurrently, and then the cells were infected with PDCoV^T-^ (MOI = 0.5). At 24 hpi, the cells were collected, and viral titers were determined by TCID_50_ (**E**) assay. Viral internalization assay was performed, and viral RNA copies were quantified by RT-qPCR (**F**). ns, no significant difference, *P* > 0.05; * *P* ≤ 0.05; ** *P* ≤ 0.01; *** *P* ≤ 0.001; **** P ≤ 0.0001.

CoVs typically enter host cells through two main pathways: endosomal fusion and plasma membrane fusion (33, 35, 43). To elucidate the entry pathways of PDCoV^T-^ in Huh7 cells, two inhibitors, E64d and AEBSF-HCl, were used to target endosomal and plasma membrane proteases, respectively. E64d is a cysteine protease inhibitor that specifically targets the active site cysteine thiol, especially inhibiting cathepsins. AEBSF-HCl is an irreversible broad-spectrum inhibitor of TTSPs. We assessed the cytotoxicity of E64d and AEBSF-HCl using CCK-8 assays, which revealed that treatment with E64d at concentrations up to 50 µM and AEBSF-HCl up to 200 µM did not significantly impact cell viability (Fig. S3A and B). Subsequently, PDCoV^T-^ (MOI = 0.5) was inoculated onto Huh7 cells pretreated with different concentrations of E64d and AEBSF-HCl, and the infectivity of PDCoV^T-^ was evaluated through IFA and TCID_50_ assays. As shown in Fig. 3A and B, reduction in PDCoV N-specific green fluorescence and lower viral titers were observed in Huh7 cells with increasing concentrations of E64d. Similarly, AEBSF-HCl treatment resulted in a dose-dependent decrease in PDCoV^T-^ infection (Fig. 3C; Fig. 3D). Furthermore, viral attachment and internalization assays indicated that the addition of E64d and AEBSF-HCl predominantly inhibited PDCoV^T-^ internalization rather than attachment (Fig. S3C through F). The combined treatment with both inhibitors led to a more pronounced reduction in PDCoV^T-^ infection and internalization efficiency, as evidenced by decreased viral titers and internalized RNA copies, respectively (Fig. 3E and F). Notably, when both inhibitors were administered concurrently, the viral titer of PDCoV^T-^ fell below the detection limit (Fig. 3E). These findings suggest that PDCoV can enter Huh7 cells via both endosomal and plasma membrane fusion pathways, and concurrent inhibition of these pathways results in a significant reduction in PDCoV infection, highlighting the critical roles of the two pathways in facilitating viral entry.

### Endosomal protease cathepsin L facilitates PDCoV infection in Huh7 cells

**Fig 4:**
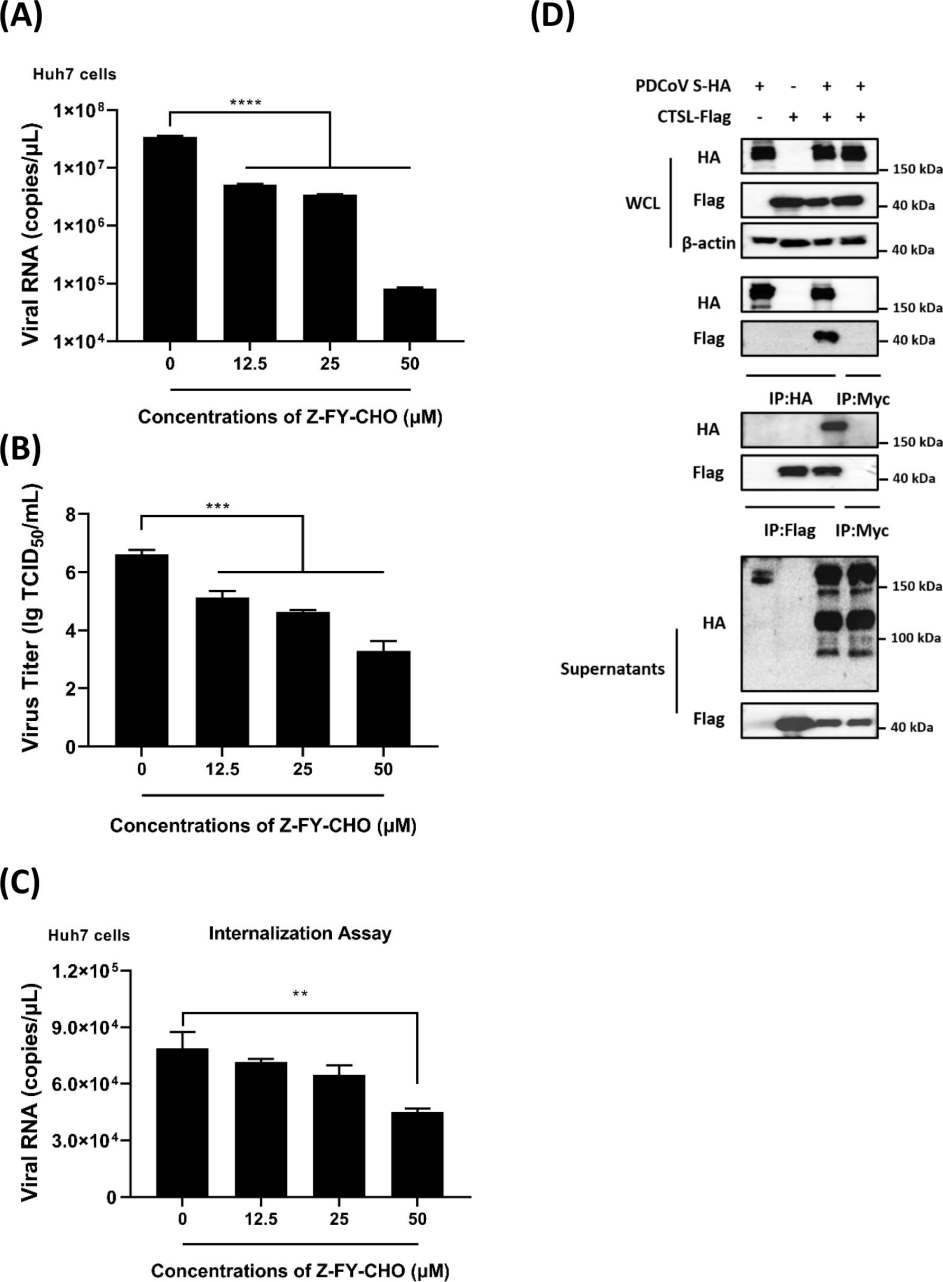
Endosomal protease cathepsin L facilitates PDCoV infection in Huh7 cells. (**A, B**) Effect of Z-FY-CHO on PDCoV infection. Huh7 cells were pretreated with different concentrations of Z-FY-CHO (0, 12.5, 25, and 50 µM), and then the cells were infected with PDCoV^T-^ (MOI = 0.5). At 24 hpi, the viral RNA copies and titers were measured by RT-qPCR (**A**) and TCID_50_ (**B**) assay, respectively. (**C**) Viral internalization assay with Z-FY-CHO. Huh7 cells were pretreated with different concentrations of Z-FY-CHO (0, 12.5, 25, and 50 µM), then the viral internalization assay was performed with PDCoV^T-^ (MOI = 1), and the internalized viral RNA copies were quantified by RT-qPCR. (**D**) Interaction and cleavage between PDCoV S and CTSL. PDCoV S and CTSL were co-transfected in HEK-293T cells, and Co-IPs were performed with the indicated antibodies. The anti-Myc tag antibodies served as negative controls. The supernatants from the cells co-expressing PDCoV S and CTSL were collected and then added with precooled acetone overnight at −20°C. After centrifugation, the resulting pellets were air-dried to remove residual acetone and then resuspended in RIPA lysis buffer for Western blot analysis. ns, no significant difference, *P* > 0.05; * *P* ≤ 0.05; ** *P* ≤ 0.01; *** *P* ≤ 0.001; **** P ≤ 0.0001.

To further identify the specific proteases involved in the endosomal pathway-mediated entry of PDCoV^T-^, the roles of cathepsin B (CTSB) and cathepsin L (CTSL), which are commonly utilized in CoV infections, were evaluated. Huh7 cells were pretreated with a CTSB-selective inhibitor (CA-074ME) or a CTSL-selective inhibitor (Z-FY-CHO), and cytotoxicity assessments demonstrated that both CA-074ME and Z-FY-CHO exhibited no detectable cytotoxicity at concentrations below 20 µM and 50 µM, respectively (Fig. S4A; Fig. 4B). Subsequently, Huh7 cells were treated with increasing concentrations of CA-074ME or Z-FY-CHO prior to infection with PDCoV^T-^ (MOI = 0.5). The results from RT-qPCR and TCID_50_ assays demonstrated that CA-074ME treatment had no significant impact on PDCoV^T-^ infection (Fig. S4B and C). In contrast, Z-FY-CHO treatment markedly reduced viral RNA copies and titers in a dose-dependent manner (Fig. 4A and B). Additionally, PDCoV^T-^ internalization assays revealed that Z-FY-CHO treatment, but not CA-074ME, significantly inhibited viral entry into Huh7 cells (Fig. 4C; Fig. S4E), further suggesting that CTSL plays a critical role in PDCoV^T-^ entry. To further explore the detailed association of virus with proteases, PDCoV S was co-transfected with CTSB or CTSL expression plasmids in HEK-293T cells. Western blot analysis showed that both CTSB and CTSL could interact with S protein (Fig. 4D; Fig. S4F), but only CTSL facilitated the cleavage of S protein (Fig. 4D). These findings suggest that CTSL may promote the cleavage of PDCoV S protein and the exposure of fusion peptide in S2 subunit, thereby mediating endosomal membrane fusion between the PDCoV envelope and endosomal membrane. Collectively, these results identify CTSL, rather than CTSB, as a key protease in the endosomal fusion pathway that activates PDCoV infection in Huh7 cells.

### Serine protease TMPRSS11E facilitates PDCoV infection in Huh7 cells

The plasma membrane fusion pathway during CoV infections is usually mediated by serine proteases such as TTSPs or exogenous trypsin (38, 40). To exclude the role of exogenous trypsin, different doses of PDCoV^T-^ were inoculated onto Huh7 cells with trypsin or in its absence. Consistent with prior findings, trypsin did not enhance the levels of PDCoV S protein in infected cells, although it did promote S protein cleavage (Fig. S5A). Furthermore, the addition of trypsin and its inhibitor, SBTI, did not result in significant changes in viral titers (Fig. S5B). After inhibiting the plasma membrane or endosomal fusion pathways using E64d, AEBSF-HCl, CA-074ME, or Z-FY-CHO, the addition of trypsin only slightly restored PDCoV^T-^ infection (Fig. S5C), further demonstrating that PDCoV^T-^ infection in Huh7 cells is independent of trypsin.

**Fig 5:**
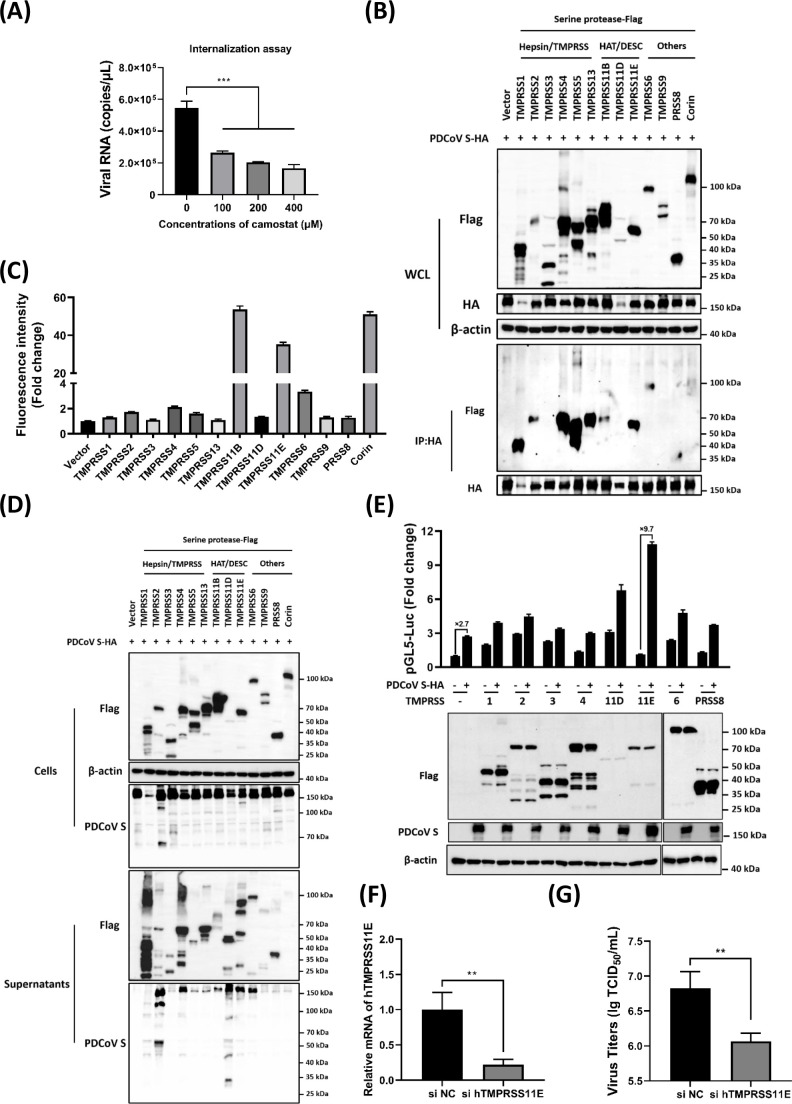
Serine protease TMPRSS11E facilitates PDCoV infection in Huh7 cells. (**A**) Viral internalization assay with camostat. Huh7 cells were pretreated with different concentrations of camostat (0, 100, 200, and 400 µM). Then, the viral internalization assay was performed with PDCoV^T-^ (MOI = 1), and the internalized viral RNA copies were quantified by RT-qPCR. (**B**) Interaction between PDCoV S and TTSPs. Expression plasmids for PDCoV S and the members of TTSP family were co-transfected in HEK-293T cells, respectively, and Co-IPs were performed with indicated antibodies. (**C**) The membrane localization analysis of TTSPs. HEK-293T cells were transfected with expression plasmids of TTSPs or empty vector for 24 h and then incubated with anti-Flag antibodies and analyzed with indirect flow cytometry. (**D**) The cleavage of PDCoV S by TTSPs. After co-expression of PDCoV S and TTSPs, the cells and supernatants were collected and subjected to Western blot analysis. (**E**) Luciferase-based cell-cell fusion assay. HEK-293T cells were co-transfected with pBind-Id, PACT-Myod, and pUC57-EF-1α-S expression plasmids or empty vector and then co-cultured with pGL5-Luc-expressing cells transfected with TTSPs. Dual-luciferase assay was performed at 24 h after co-culture. The expressions of TTSPs, PDCoV S, and β-actin were detected via Western blot assay. β-actin served as a protein loading control. (**F, G**) Effect of interference with TMPRSS11E on PDCoV^T-^ proliferation. Huh7 cells were transfected with specific siRNA targeting TMPRSS11E, and after 24 h, the mRNA levels of TMPRSS11E were detected by RT-qPCR (**F**). Then, PDCoV^T-^ was inoculated on Huh7 cells, and viral titers were determined at 12 hpi by TCID_50_ assay (**G**). ns, no significant difference, *P* > 0.05; * *P* ≤ 0.05; ** *P* ≤ 0.01; *** *P* ≤ 0.001; **** P ≤ 0.0001.

Next, we investigated the involvement of TTSPs using camostat, a surface serine protease inhibitor known to inhibit the activity of nearly 20 members of the TTSP family. Viral internalization assay showed that camostat pretreatment reduced viral entry efficiency in a dose-dependent manner (Fig. 5A), providing preliminary evidence that TTSPs contribute to PDCoV^T-^ infection in Huh7 cells. Subsequently, the expression plasmids of 14 TTSPs were constructed and co-transfected with a plasmid encoding the PDCoV S protein. The results of co-immunoprecipitations (Co-IPs) revealed that several TTSPs (including TMPRSS1, TMPRSS2, TMPRSS4, TMPRSS5, TMPRSS13, TMPRSS11B, TMPRSS11E, TMPRSS6, and PRSS8) interacted with the S protein (Fig. 5B). Given that TTSP-mediated cleavage of the S protein typically occurs at the cell membrane, we also analyzed the membrane localization of these 14 members using indirect flow cytometry. Notably, TMPRSS11B, Corin, TMPRSS11E, and TMPRSS6 exhibited significant localization at the cell membrane (Fig. 5C). Further analyses of both cell lysates and supernatants identified TMPRSS1, TMPRSS2, TMPRSS3, TMPRSS4, TMPRSS11D, TMPRSS11E, TMPRSS6, and PRSS8 as the primary proteases responsible for cleaving the PDCoV S protein (Fig. 5D). Additionally, the efficiency of membrane fusion activated by S protein cleavage was evaluated, and dual-fluorescence assays demonstrated that TMPRSS11E overexpression increased fluorescence ratios, thereby enhancing PDCoV S protein-mediated membrane fusion (Fig. 5E). Furthermore, when endogenous TMPRSS11E expression in Huh7 cells was knocked down using specific siRNA (Fig. 5F), the viral titers were significantly decreased after PDCoV^T-^ infection (Fig. 5G). Collectively, these findings indicate that TTSPs, particularly TMPRSS11E, play a crucial role in facilitating PDCoV entry and infection in Huh7 cells.

### Furin promotes the maturation and release of PDCoV virion in Huh7 cells

**Fig 6:**
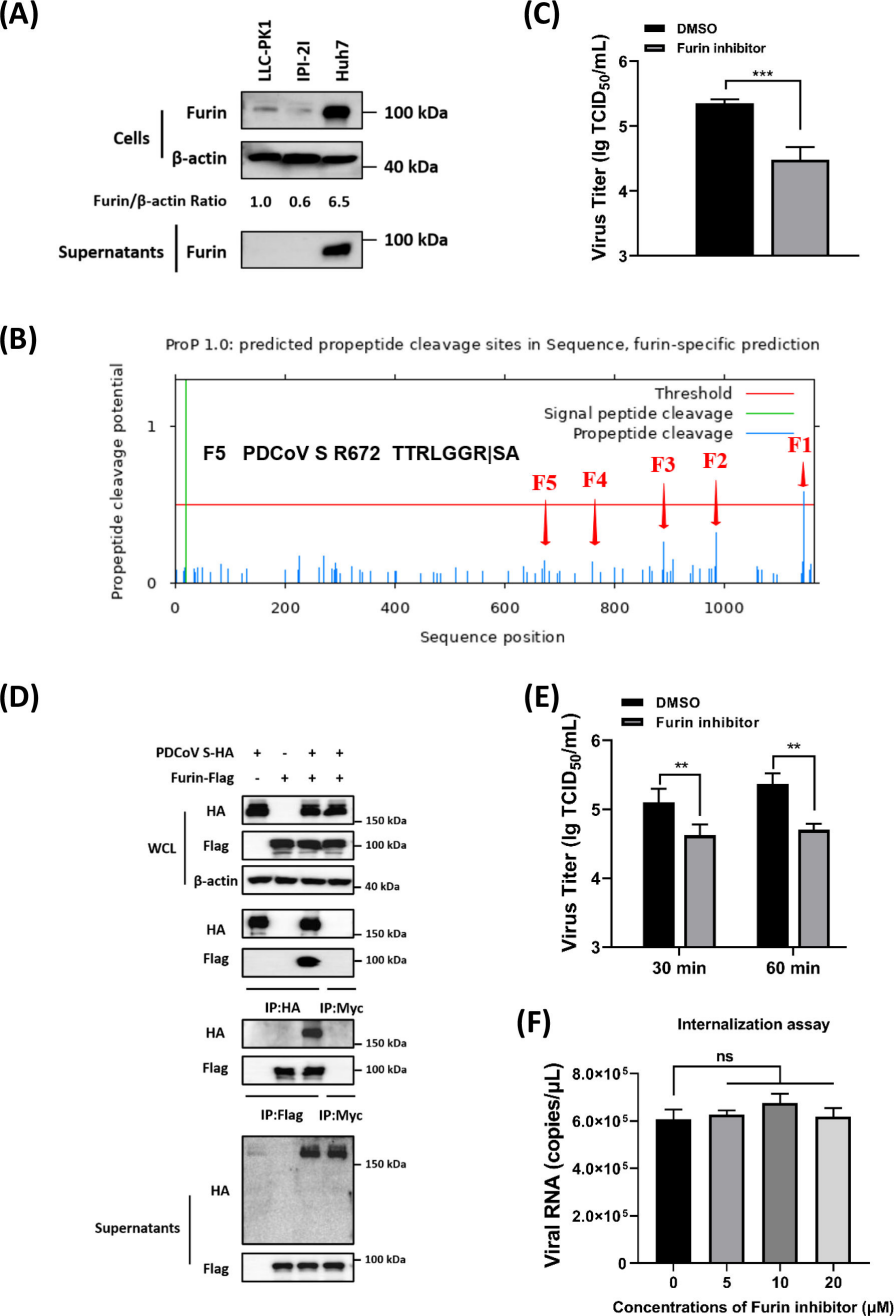
Furin promotes the release of PDCoV virions in Huh7 cells. (**A**) The abundance of endogenous furin protein was analyzed in PDCoV-susceptible cell lines, including LLC-PK1, IPI-2I, and Huh7 cells. The expression of the target proteins was detected by Western blot and quantitatively estimated with ImageJ software, and the numbers below the images represent the relative levels of furin/β-actin. (**B**) Analysis of the furin cleavage sites on PDCoV S protein via a ProP software (http://www.cbs.dtu.dk/services/ProP/). (**C**) The effect of furin inhibitor on PDCoV^T-^ infection. Huh7 cells were pretreated with a furin inhibitor (20 µM), and then the cells were infected with PDCoV^T-^ (MOI = 0.5). At 12 hpi, viral titers were measured by TCID_50_ assay. (**D**) The interaction and cleavage between PDCoV S protein and furin. Expression plasmids for PDCoV S protein and furin were co-transfected into HEK-293T cells, and then Co-IPs were performed with indicated antibodies. The anti-Myc tag antibodies served as negative controls. The supernatants of cells co-expressing PDCoV S protein and furin were collected and then added with precooled acetone overnight at −20°C. After centrifugation, the resulting pellets were air-dried to remove residual acetone and then resuspended in RIPA lysis buffer for Western blot analysis. (**E**) Viral release assay. Huh7 cells were infected with PDCoV^T-^ at an MOI of 0.5. At 10 hpi, the medium was replaced with fresh DMEM with or without furin inhibitor. The supernatants were collected at 30 and 60 min, and viral titers were determined by TCID_50_ assay. (**F**) Viral internalization assay with furin inhibitor. Huh7 cells were pretreated with different concentrations of furin inhibitor (0, 5, 10, and 20 µM), then the viral internalization assay was performed with PDCoV^T-^ (MOI = 1), and the internalized viral RNA copies were quantified by RT-qPCR. ns, no significant difference, *P* > 0.05; * *P* ≤ 0.05; ** *P* ≤ 0.01; *** *P* ≤ 0.001; **** P ≤ 0.0001.

Furin is ubiquitously expressed in mammalian cells, but its expression levels are typically low under normal conditions (44). A previous study reported that the levels of furin protein are relatively high in Huh7 cells (45), suggesting that the viral activation mechanism may differ in cells with high furin expression. Consistently, endogenous detection confirmed that the expression levels of furin in Huh7 cells were higher than those in IPI-2I and LLC-PK1 cells, two PDCoV-susceptible porcine-derived cell lines (Fig. 6A). Additionally, the results demonstrated that furin was prominently detected in the culture supernatants of Huh7 cells, whereas no detectable furin secretion was observed in either LLC-PK1 or IPI-2I cells (Fig. 6A). According to predictions from the ProP software (http://www.cbs.dtu.dk/services/ProP/), the PDCoV S protein contains several potential furin cleavage sites, with the highest-scoring site being F1 (0.58) at LFSKKKR↓YT (residues 1137–1146) (Fig. 6B). Although the F5 site at TTRLGGR↓SA (residues 666–674) has a relatively low score (0.14) (Fig. 6B), it represents a potential S2’ cleavage site of the PDCoV S protein, and the high furin activity in Huh7 cells may facilitate sufficient cleavage at this site. To validate this hypothesis, Huh7 cells were treated with a specific furin inhibitor, and a significant reduction in viral titers was observed during PDCoV^T-^ infection (Fig. 6C). Additionally, exogenous interaction between furin and PDCoV S protein was examined. The results revealed that furin did not produce detectable S protein cleavage fragments (Fig. 6D); it significantly increased the levels of S protein in the supernatants, indicating that furin promotes the secretion of the S protein, thereby accelerating the release of PDCoV^T-^ virions. To verify this hypothesis, a furin inhibitor was added at the release stage of PDCoV^T-^, and viral titers in supernatants were evaluated. The results showed that the addition of the inhibitor significantly reduced viral titers in the supernatants (Fig. 6E), indicating that furin plays a critical role in the release of PDCoV^T-^ virions. However, the inhibitor did not affect viral internalization into the cells (Fig. 6F). Collectively, these findings confirmed that furin contributes to the later stages of viral maturation and release, rather than the early stages of viral entry.

## DISCUSSION

CoVs often utilize different strategies to facilitate infection in various tissue or species, and the tropism is commonly determined by the distribution of cellular receptors (43, 46). When the receptor expression is ubiquitous, the abundance of proteases in various tissues or cells becomes the critical determinant of viral tropism (39, 47). For instance, both SARS-CoV and SARS-CoV-2 bind to the ACE2 receptor, which is broadly expressed in tissues such as the lungs, heart, kidneys, and intestines (48, 49). However, the effective infections of SARS-CoV or SARS-CoV-2 necessitate the cleavage of the S protein by host proteases, such as TMPRSS2 (27, 36). TMPRSS2 is highly expressed in lung epithelial cells, making these cells the primary target for SARS-CoV and SARS-CoV-2 infections (27, 50, 51). Similarly, MERS-CoV binds to the DPP4 receptor, and its tissue susceptibility is closely correlated with the abundant expression of furin in the respiratory and gastrointestinal tracts (52, 53). As for PDCoV infection, our previous study demonstrated that PDCoV infection in porcine-derived cells is primarily mediated by trypsin, consistent with PDCoV susceptibility in the gastrointestinal tracts of piglets (41). In this study, we revealed distinct protease dependencies between human- and porcine-derived cells, suggesting different infection mechanisms of PDCoV entry in different species. In human-derived cells, PDCoV infection is trypsin-independent but relies on two alternative proteases: the cysteine protease CTSL (Fig. 4) and the serine protease TMPRSS11E (Fig. 5). CTSL is highly expressed in the placenta, appendix, and lung tissue (54), while TMPRSS11E expression is restricted to the epithelia of the skin and oral cavity (55). PDCoV infections in humans tend to present milder symptoms and have not been extensively reported (22). The localization of these proteases may contribute to the infection discrepancies observed between humans and pigs.

**Fig 7:**
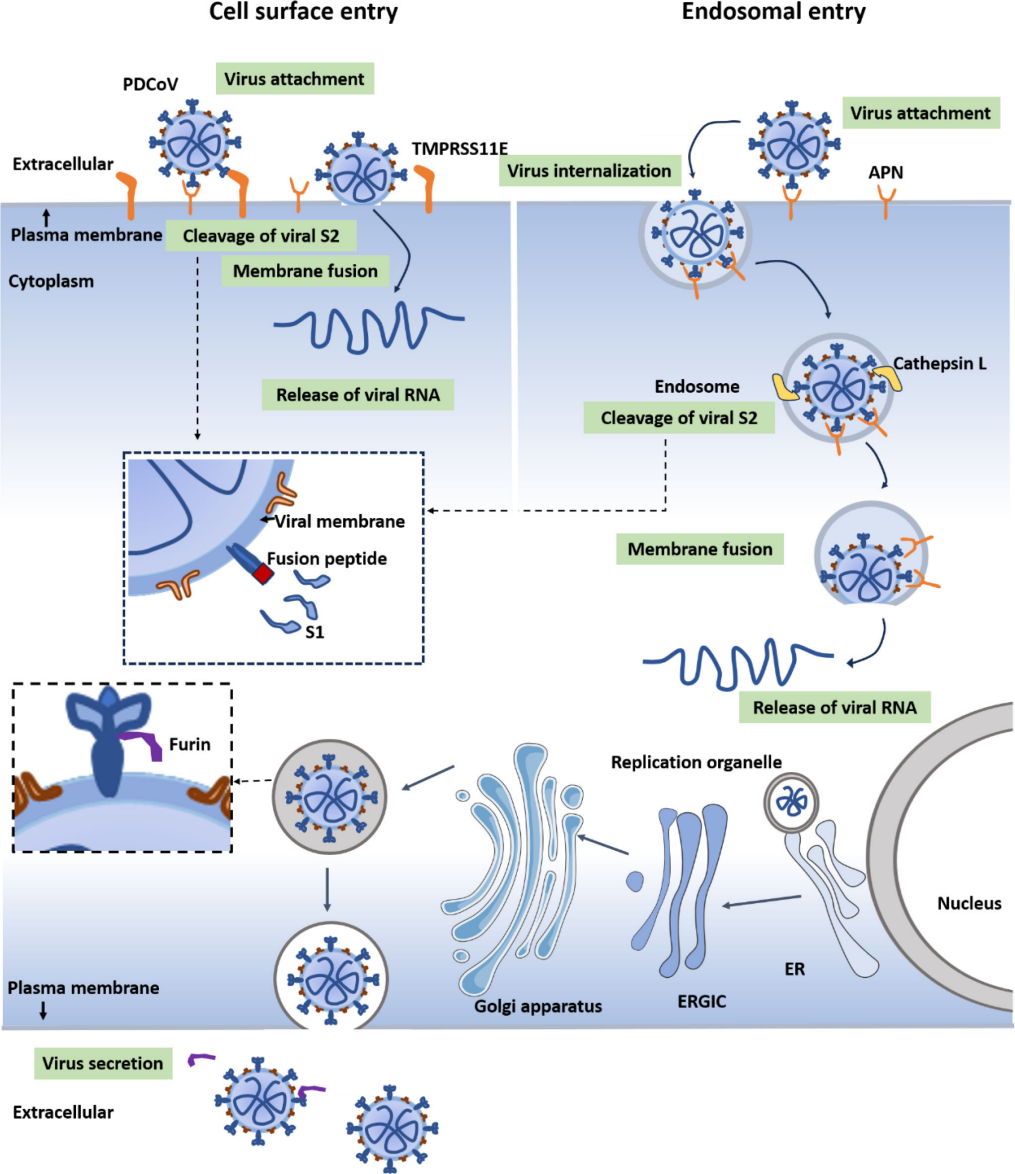
A working model illustrating how PDCoV enters and releases in human-derived cells. PDCoV enters human-derived cells through a series of coordinated steps involving receptor binding, membrane fusion, and the release of the viral genome. Initially, the S1 subunit of the S protein binds to the cellular receptor APN, triggering conformational changes in the S protein. Subsequently, PDCoV enters the cell via two distinct fusion pathways: (i) the CTSL-mediated endosomal fusion pathway, which facilitates fusion of the viral envelope with the endosomal membrane, and (ii) the TMPRSS11E-mediated plasma membrane fusion pathway, which enables the fusion of the viral envelope with the cell membrane. Following entry, the nucleocapsid is released into the cytoplasm, where uncoating occurs, exposing the viral genome. The viral RNA is subsequently transcribed and translated to produce structural and nonstructural proteins. The S, E, and M proteins are inserted into the endoplasmic reticulum and trafficked to the ER-Golgi intermediate compartment (ERGIC). The N protein encapsidates the newly synthesized positive-sense RNA genome to form the nucleocapsid. The nucleocapsid buds into the ERGIC, acquiring its envelope and spike structure to form mature virions. Finally, the assembled virions undergo furin-mediated release from the human-derived cells.

CoVs usually enter host cells through endosomal or plasma membrane fusion pathways or a combination of both. PDCoV infection in porcine-derived cells is significantly impeded in the absence of trypsin, indicating a marked dependence on the plasma membrane fusion pathway. Although some studies have reported low expression levels of TTSPs in Huh7 cells (56), PDCoV can still efficiently infect human-derived cells without the supplementation of exogenous trypsin (Fig. 1). These findings suggest that an increased dependence of PDCoV on the endosomal fusion pathway for the infection in human-derived cells (Fig. 7). Consistently, PDCoV replication was reduced to undetectable levels only when inhibitors targeting both endosomal and plasma membrane fusion pathways were applied simultaneously (Fig. 3E). CTSL has been well-characterized as a crucial host protease facilitating the endosomal entry pathway of multiple coronaviruses, including SARS-CoV, MERS-CoV, and SARS-CoV-2 (57, 58). It represents a promising therapeutic target for COVID-19; however, current evidence primarily stems from *in vitro* and preclinical studies (59, 60). SARS-CoV-2 primarily utilizes plasma membrane fusion as its default entry pathway; however, it can resort to the endosomal fusion pathway in the absence of TMPRSS2, particularly in cells lacking TMPRSS2 expression (61). Similar to TMPRSS2, TMPRSS11E (also known as DESC1) belongs to the TTSPs family, yet the two proteins exhibit differences in tissue distribution and substrate preferences. Recent studies have identified TMPRSS11E as an alternative activator for influenza virus and some coronaviruses in respiratory epithelial cells (62). Therefore, for TMPRSS2-independent coronaviruses, other members of the TTSP family may functionally compensate during the entry of PDCoV. These observations underscore the significance of the microenvironment in modulating the entry pathway, highlighting the adaptability of CoVs in optimizing their infection strategies.

Furin is a proprotein convertase involved in a variety of physiological and pathological processes (63, 64). Notably, furin has been confirmed to facilitate viral infections by cleaving the glycoproteins, including those of HIV-1, measles virus, influenza virus, and some CoVs (39, 65, 66). Furin activates the cleavage of the S protein of CoVs, facilitating viral entry, as observed in the case of MERS-CoV, which contains a classical furin cleavage site (R-X-K/R-R) within the S1/S2 loop (52). Recent studies have demonstrated that deletion of the furin cleavage site reduces SARS-CoV-2 replication in human respiratory epithelial cells and attenuates pathogenesis in hamster and mouse models (67–69). Kathryn et al. introduced a furin cleavage site at the putative S1/S2 junctional region of the SARS-CoV S protein. The cleavage of this site did not affect viral entry but cell-cell fusion (70). These studies highlight the critical role of furin in the infection of CoVs. In this study, treatment with a specific furin inhibitor significantly reduced the release of PDCoV (Fig. 6E), whereas no substantial impact was observed on viral entry (Fig. 6F). Regarding the furin site recognition, the double glycine motif (RLGGR↓) may contribute to the poor ProP prediction score, exhibiting no detectable cleavage fragments of S protein. Furthermore, PDCoV infection was carried out with a furin inhibitor in LLC-PK1 cells, which exhibit relatively low endogenous furin expression and no furin secretion compared to Huh7 cells. The results showed that treatment with the furin inhibitor did not significantly reduce PDCoV titers in LLC-PK1 cells (data not shown), in contrast to the observations made in Huh7 cells (Fig. 6C). Therefore, these results suggested that furin may facilitate the migration of PDCoV S protein through its interaction with S protein, indicating that furin dependency may be cell-type-specific, correlating with the basal expression levels present in different cell lines. Based on these results, furin inhibitor treatment decreased viral titers during the release stages through potential mechanisms: (i) low-efficiency cleavage of PDCoV S, leading to the active trafficking of S protein and virions; (ii) interaction between furin and PDCoV S protein, where furin secretion facilitates the passive migration of S protein and virions (Fig. 7).

The utilization of proteases by CoVs involves multifaceted mechanisms (71). In addition to the commonly recognized localization on the cell membrane, our study revealed that proteases can also be secreted into the cell culture supernatants (Fig. 5D and 6A). These proteases in the supernatants act as substitutes for exogenous trypsin in promoting viral entry (Fig. 2E through G). However, the addition of culture supernatants from Huh7 cells resulted in only a limited increase of PDCoV infection on LLC-PK1 cells under trypsin-free conditions (Fig. 2E through G). These discoveries suggest that proteases in the supernatants may mainly contribute to the initial viral entry, whereas the subsequent viral replication and productive infection require the involvement of intracellular proteases, such as CTSL and/or furin. Therefore, these findings suggest that proteases are not only essential for viral entry but also play significant roles in the later stages during the viral replication cycle, highlighting the cooperative roles of different proteases in the intricate infection mechanisms of CoVs.

In summary, our findings demonstrate that PDCoV enters human-derived cells through both endosomal and plasma membrane fusion pathways (Fig. 7). Specifically, the endosomal pathway is mediated by CTSL, and TMPRSS11E facilitates the virus-cell fusion at the plasma membrane. These proteases promote the cleavage of the PDCoV S protein, thereby activating viral entry. Furthermore, furin is confirmed to play a crucial role in the maturation and secretion of virions. This study provides detailed insights into the infection mechanisms of PDCoV in human-derived cells.

## MATERIALS AND METHODS

### Cells, viruses, and antibodies

Huh7 (human hepatocyte carcinoma), HEK-293T (human embryonic epithelial kidney), HeLa (human cervical adenocarcinoma), HepG2 (human liver cancer) cells and IPI-2I (porcine ileum epithelial) cells were cultured in Dulbecco’s modified Eagle medium (DMEM, Gibco, USA). LLC-PK1 (porcine kidney) cells were cultured in minimum essential medium (MEM, Hyclone, USA). A549 (human lung adenocarcinoma epithelial) cells were cultured in DMEM/Nutrient Mixture medium (DMEM/F-12, Hyclone, USA). All media were supplemented with 10% FBS (TransGen, China) and 1% penicillin-streptomycin solution (Hyclone), and cells were cultured at 37°C with 5% CO_2_. PDCoV strain CHN-HN-2014 (GenBank accession number KT336560) was propagated in Huh7 or LLC-PK1 cells and then titrated on LLC-PK1 cells in MEM supplemented with 7.5 µg/mL trypsin. Mouse anti-PDCoV N and anti-PDCoV M monoclonal antibodies (mAbs) were prepared by our laboratory. Rabbit anti-PDCoV S polyclonal antibodies (pAbs) were prepared by DIA-AN (China) using purified recombinant S protein. Mouse anti-Flag and anti-HA mAbs were purchased from Cell Signaling Technology (CST, USA). Rabbit anti-β-actin pAbs were purchased from Abclonal (China). Rabbit anti-furin pAbs were purchased from Proteintech (China).

### RNA extraction and reverse transcription-quantitative PCR (RT-qPCR)

Cellular total RNA was extracted at indicated hours post-infection (hpi) using TRIzol reagent (Invitrogen, USA) following the manufacturer’s protocol. After the RNA concentration and purification were checked, 1 µg of total RNA was subsequently retro-transcribed into cDNA with the HiScript II Q RT SuperMix (Vazyme, China) according to the manufacturer’s instructions. RT-qPCR assays were performed to quantify gene expression using the HiScript II one-step qRT-PCR SYBR green kit (Vazyme, China). Reactions were carried out in a 10 µL final volume on an ABI 7500 real-time PCR system (Applied Biosystems, USA) under the following conditions: an initial denaturation step at 95°C for 5 minutes, followed by 40 cycles of denaturation at 95°C for 15 seconds, annealing at 58°C for 30 seconds, and extension at 72°C for 30 seconds. Fluorescence was captured at the end of each amplification cycle. The specific primers used for target genes are as follows: PDCoV N, forward, 5′-AGCTGCTACCTCTCCGATTC-3′, reverse, 5′-ACATTGGCACCAGTACGAGA-3′; TMPRSS11E, forward, 5′-ATTGTCCTGGCAGTGTGCA-3′, reverse, 5′- AAGCCTCTCTGCCAAACTCA-3′; GAPDH, forward, 5′- ATGGCAAATTCCATGGCACC-3′, reverse, 5′-TCGCCCCACTTGATTTTGGA-3′. Viral RNA copy numbers were determined by the absolute quantification method with a standard curve generated from serially diluted PDCoV N gene plasmid. The relative mRNA levels of TMPRSS11E were evaluated by the relative quantification method, with GAPDH as a reference gene.

### 50% tissue culture infective dose (TCID_50_) assay

Viral titers in PDCoV-infected cells were determined by TCID_50_ assay on LLC-PK1 cells. Briefly, a monolayer of LLC-PK1 cells in 96-well plates was washed twice with MEM containing 7.5 µg/mL trypsin, then the viral samples were serially 10-fold diluted, and 100 µL of each dilution was added into wells of the 96-well plate in octuplicate. After incubation at 37°C for 2–3 days, cytopathic effects (CPE) were continuously monitored until no further increase in wells containing CPE was observed. The viral titers were calculated using the Reed-Muench method and expressed as TCID_50_ per milliliter (TCID_50_/mL).

### Indirect immunofluorescence assay (IFA)

Cells cultured in 24-well plates were inoculated with PDCoV^T-^ when the cells reached 100% confluence. At the indicated hpi, the cells were fixed with 4% paraformaldehyde for 15 min and then permeated with precooled methyl alcohol for 10 min at room temperature. After being washed three times with phosphate-buffered saline (PBS, pH = 7.4), the cells were blocked with 5% bovine serum albumin (BSA) for 1 h at 37°C. Primary antibodies specific to the PDCoV N were diluted in the blocking solution, followed by incubation for 1 h at 37°C. After washing three times with PBS to remove unbound primary antibodies, the cells were subsequently incubated with the secondary antibody for 1 h at 37°C and 4ʹ, 6-diamidino-2-phenylindole (DAPI; Beyotime, China) for 10 min at room temperature. After the samples were washed three times with PBS, fluorescent images were captured with a fluorescence microscope (Olympus).

### Viral attachment, internalization, and release assays

Confluent cells cultured in 6-well plates were precooled at 4°C for 30 min, and the medium was then replaced with the mixture of trypsin and PDCoV^T-^ (MOI = 1). After incubation at 4°C for 1 h, the cells were washed three times with PBS to remove the unbound virus. The attached PDCoV RNA was then quantified by RT-qPCR. For the viral internalization assay, after viral attachment, the medium was replaced by fresh medium containing different concentrations of trypsin, and the cells were cultured at 37°C for another 1 h. After washing three times with citrate buffer (50 mM citrate, 50 mM sodium dihydrogen citrate, pH 3.0) to remove the extracellular virus, the internalized PDCoV^T-^ RNA was quantified by RT-qPCR. The viral RNA copies detected in groups without trypsin pretreatment were set as 1. For viral release assay, cells were infected with PDCoV^T-^ (MOI = 0.5). At 10 hpi, the medium was replaced by fresh medium, and the supernatants were collected at 30 and 60 min, and viral titers were measured by TCID_50_ assay.

### Co-immunoprecipitation (Co-IP) and Western blot

HEK-293T cells were transiently transfected with the indicated plasmids using jetPRIME transfection reagent (Polyplus, France) following the manufacturer’s protocol. After 24 h of transfection, the cells were harvested and then lysed with Co-IP buffer. The whole-cell lysates (WCL) were centrifuged and then incubated with primary antibodies at 4°C for 8 h with gentle agitation. Protein A + G agarose beads (Beyotime, China) were then added into the antibody-protein mixture and incubated for another 4 h. After incubation, the beads were washed three times with Co-IP buffer, and the immunoprecipitated proteins were eluted by boiling the beads in 1 × SDS loading buffer for 10 min. As for protein detection in the supernatants, the supernatants were collected and precipitated with acetone at −20°C, followed by dissolving in lysis buffer. Next, the samples were separated by SDS-PAGE and transferred to polyvinylidene difluoride membranes (PVDF, Millipore, USA). These membranes were blocked with TBST supplemented with 10% skim milk for 2 h at room temperature. After blocking, the membranes were incubated with the primary antibodies for 4 h at room temperature or overnight at 4°C, followed by incubation with horseradish peroxidase (HRP)-conjugated secondary antibodies (Beyotime, China) for 1 h at room temperature. Finally, the bands were visualized using an enhanced chemiluminescence (ECL) detection reagent (Bio-Rad, USA) and captured using a chemiluminescence imaging system.

### Cytotoxicity analysis and drug treatment

Cytotoxicity of the drugs was assessed using the cell-counting kit-8 (CCK-8) assay (Beyotime, China). Briefly, Huh7 cells seeded in 96-well plates were treated with different concentrations of the indicated drugs for 24  h. Following treatment, 10 µL of the CCK-8 reagent was added to each well, and the cells were incubated for 2 h at 37°C according to the manufacturer’s instructions. The absorbance at 450 nm was measured with a microplate spectrophotometer (Cytation, Biotek, USA) at 37°C. Cell viability was calculated as a percentage relative to untreated controls. To evaluate the effects of drugs on the PDCoV^T-^ entry or infection, confluent cells were pretreated with the indicated concentrations of E64d (MCE, USA), AEBSF-HCl (Sigma, USA), CTSB-selective inhibitor (CA-074ME, MCE, USA), CTSL-selective inhibitor (Z-FY-CHO, MCE, USA), camostat (MCE, USA), or Furin inhibitor (Sigma, USA) for 1  h at 37°C. Then, cells were infected with PDCoV^T-^ in the continued presence of the drugs. At the indicated hpi, cells were harvested for next analyses, including quantification of viral RNA by RT-qPCR and determination of viral titers by TCID_50_ assay.

### Cell-cell fusion assay

To evaluate cell-cell fusion quantitatively, a luciferase-based cell-cell fusion assay was performed as previously described (72). Briefly, donor cells were seeded in six-well plates and co-transfected with 1 µg reporter plasmid pGL5-luc, 0.1 µg of the internal control plasmid pRL-TK, and 1 µg of the expression plasmid pCAGGS-TTSPs-Flag. Target cells were seeded in six-well plates and co-transfected with 1 µg pBind-Id, 1 µg PACT-Myod, and 1 µg PDCoV S-expressing plasmid or empty vector control. At 24 h post-transfection, donor and target cells were cocultured at a 1:1 ratio and then co-cultured in fresh medium for 24 h to allow for cell-cell fusion. Then, the cells were lysed, and the activities of firefly luciferase and Renilla luciferase were measured using a dual-luciferase reporter assay system (Promega, USA). Cell-cell fusion activity was determined by calculating the ratio of firefly luciferase activity to Renilla luciferase activity, and data were normalized to the activity observed in cells transfected with the empty vector control.

### Small interfering RNA (siRNA) transfection

siRNA targeting TMPRSS11E (si-TMPRSS11E: 5′-GCUGUGGAGCAACCUUAAUTT-3′) and the negative control siRNA (si-NC: 5'- UUCUCCGAACGUGUCACGUTT-3′) were synthesized and purchased from Genepharma (China). Huh7 cells were seeded in appropriate culture plates and transfected with siRNA at a final concentration of 50 nM according to the manufacturer’s instructions. After 24 h, the cells were infected with PDCoV^T-^ (MOI = 0.5) and harvested at indicated hpi for subsequent analyses, including mRNA quantification by RT-qPCR and viral titration assays.

### Statistical analyses

All data are presented as mean ± standard deviation (SD). Statistical differences are analyzed using one-way ANOVA or Student’s *t*-test with GraphPad Prism 8.0 software. Significant differences are indicated by asterisks as follows: ns, no significant difference, *P* > 0.05; * *P* ≤ 0.05; ** *P* ≤ 0.01; *** *P* ≤ 0.001; **** P ≤ 0.0001.

## Data Availability

All methods and data described in this article are available from the corresponding author upon request.

## SUPPLEMENTAL MATERIAL

Supplemental figures jvi.01055-25-s0001.docx Fig. S1 to S5.

ASM does not own the copyrights to Supplemental Material that may be linked to, or accessed through, an article. The authors have granted ASM a non-exclusive, world-wide license to publish the Supplemental Material files. Please contact the corresponding author directly for reuse.
