# Supplementary material for: Cathepsin L and transmembrane serine protease 11E mediate trypsin-independent entry of porcine deltacoronavirus into Huh7 cells
Source: J Virol. 2025 Aug 11;99(9):e01055-25. doi: 10.1128/jvi.01055-25 (PMC12456011; doi:10.1128/jvi.01055-25)
Supplement: Supplemental figures — Fig. S1 to S5. [file jvi.01055-25-s0001.docx]

**Supplemental Materials**

**Fig. S1.** Exogenous trypsin has no significant effect on PDCoV infection in multiple human-derived cells.

**Fig. S2.** Purification of PDCoV^T-^ virions from LLC-PK1 and Huh7 cells.

**Fig. S3.** Cytotoxicity of E64d and AEBSF-HCl and their effects on PDCoV entry into Huh7 cells.

**Fig. S4.** Endosomal protease cathepsin B does not facilitates PDCoV infection in Huh7 cells.

**Fig. S5.** PDCoV infection in Huh7 cells is independent of trypsin activity.

**
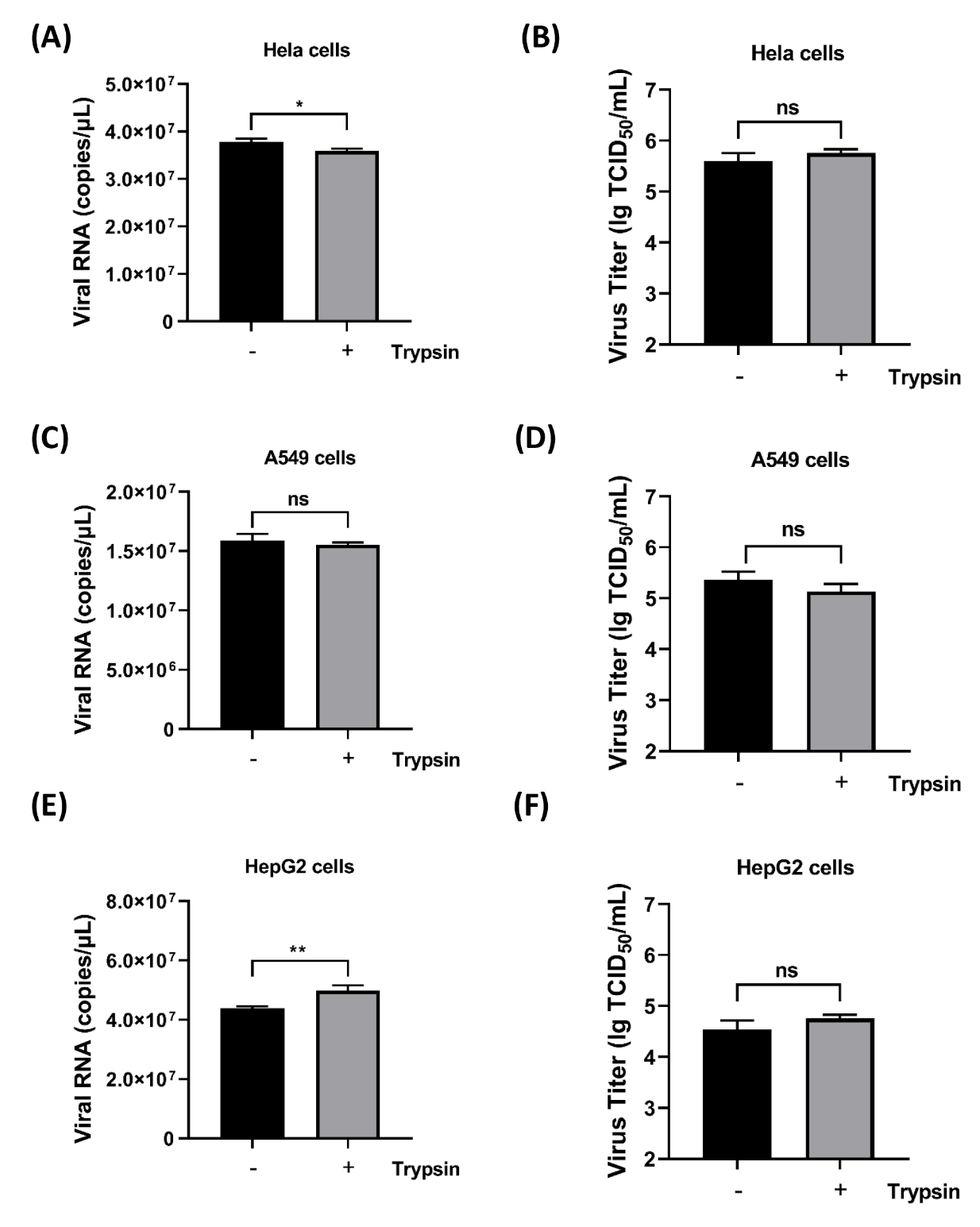
Fig. S1**

**Fig. S1. Exogenous trypsin has no significant effect on PDCoV infection in multiple human-derived cells.** PDCoV^T-^ (MOI=1) was inoculated on Hela (A, B), A549 (C, D) and HepG2 (E, F) cells with or without trypsin (1 μg/mL). At 24 hpi, the cells were harvested, viral RNA copies and titers were measured by RT-qPCR (A, C, E) and TCID_50_ assays (B, D, F), respectively.

**
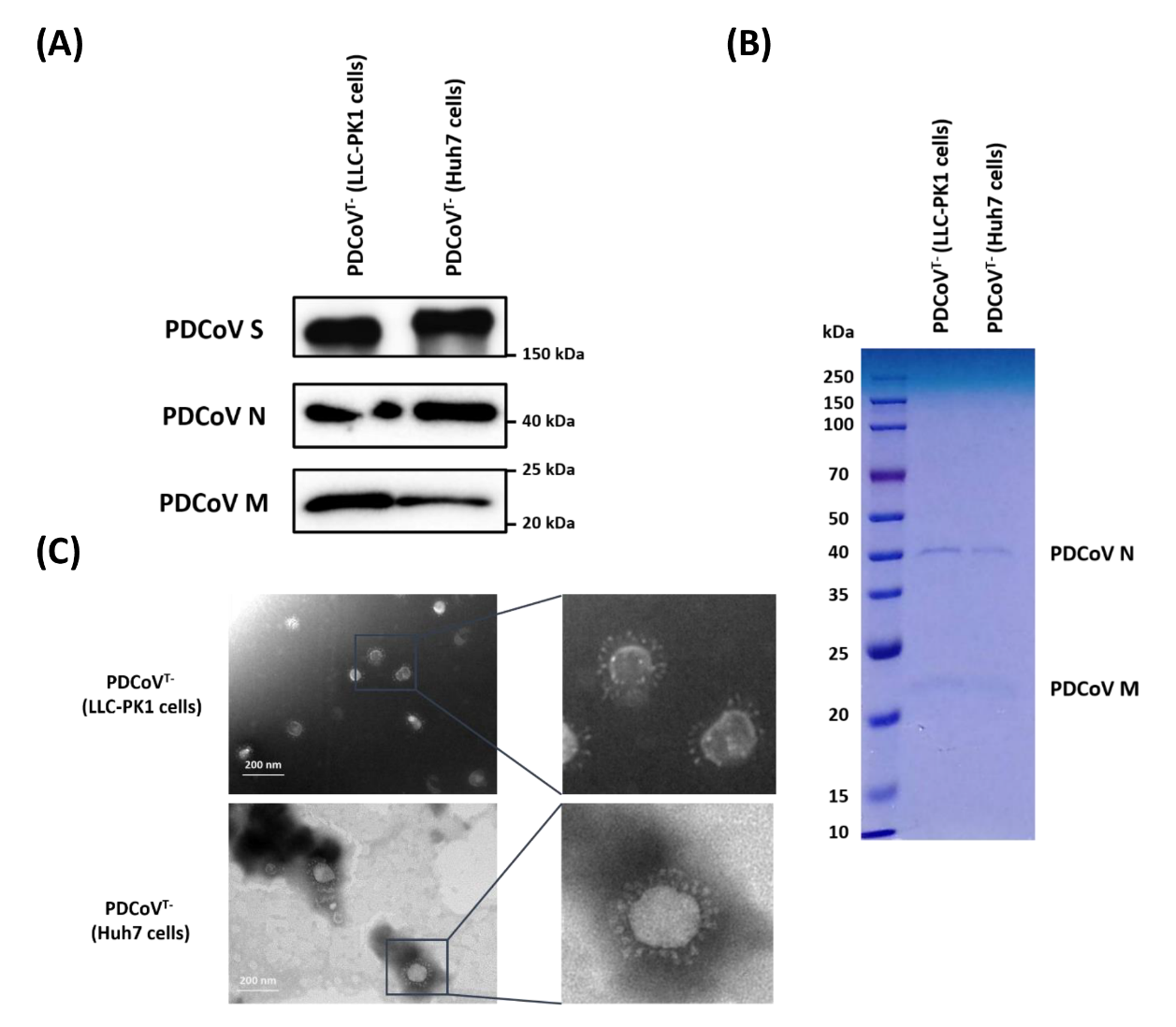
Fig. S2**

**Fig. S2. Purification of PDCoV^T-^ virions from LLC-PK1 and Huh7 cells.** The culture supernatants of PDCoV^T-^-infected LLC-PK1 and Huh7 cells were collected and concentrated with ultrafiltration tube. Then the virions were purified using Capto Core 700. The structure proteins of PDCoV virions were detected by western blot (A) and coomassie brilliant blue staining (B). The morphological characteristics of PDCoV virions were observed via transmission electron microscopy (C) analyses. Scale bar, 200 nm.

**Fig. S3**

**
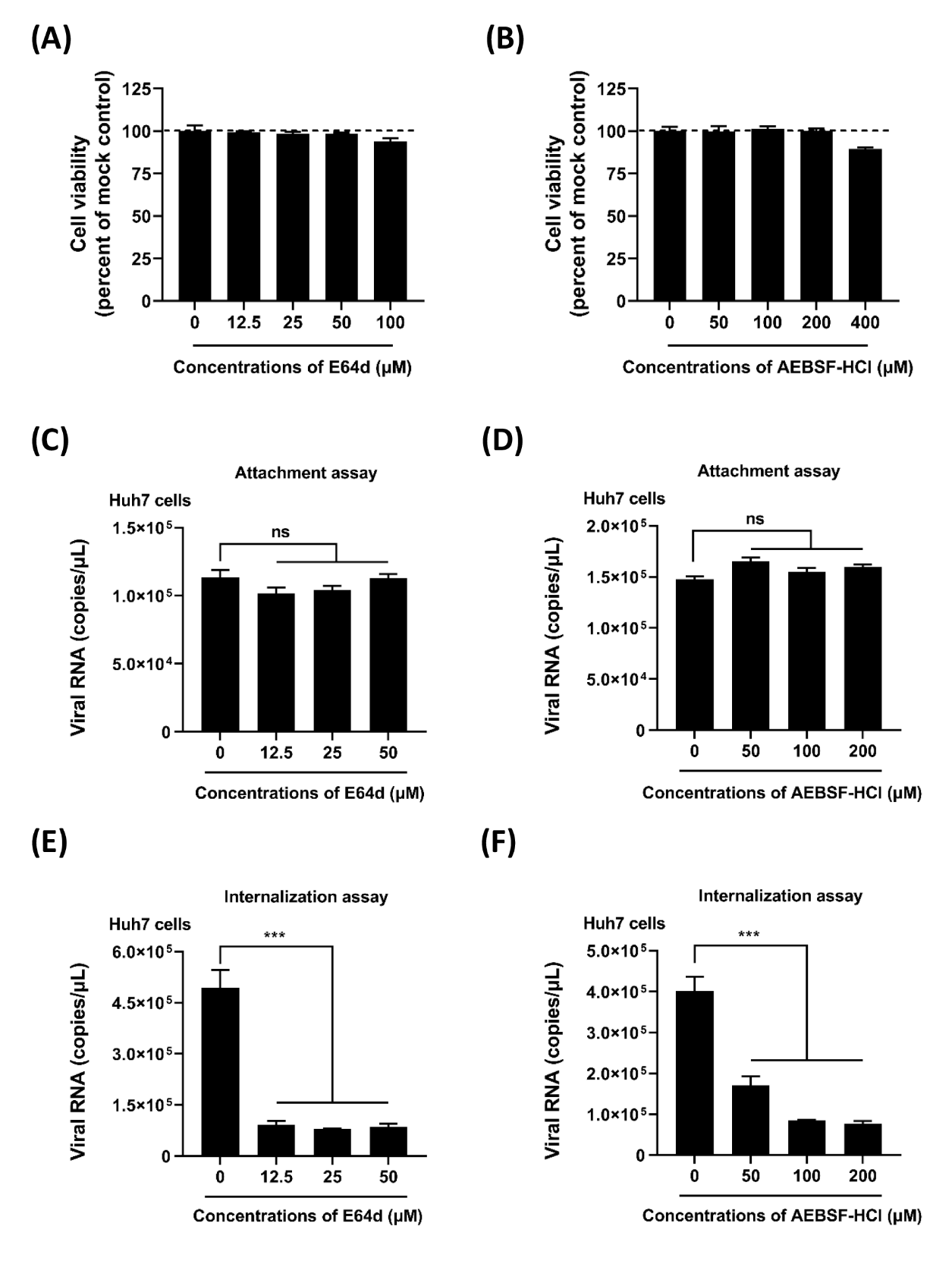
**

**Fig. S3. Cytotoxicity of** **E64d and AEBSF-HCl and their** **effects on PDCoV entry into Huh7 cells. (A, B)** The cytotoxicity of E64d and AEBSF-HCl. Huh7 cells were treated with 0-100 μM of E64d (A) or 0-400 μM of AEBSF-HCl (B) for 24 h, followed by a CCK-8-based cell viability assay in accordance with the manufacturer’s protocols. **(C-F)** Viral attachment (C, D) and internalization (E, F) on Huh7 cells. Huh7 cells were pretreated with different concentrations of E64d (C, E) or AEBSF-HCl (D, F), then infected with PDCoV^T-^ (MOI=1) at 4℃ (attachment) or 37℃ (internalization) for 1 h. After viral attachment or internalization phases, cells were washed with precool PBS or citric acid, respectively. The attached and internalized viral RNA copies were quantified by RT-qPCR.

**
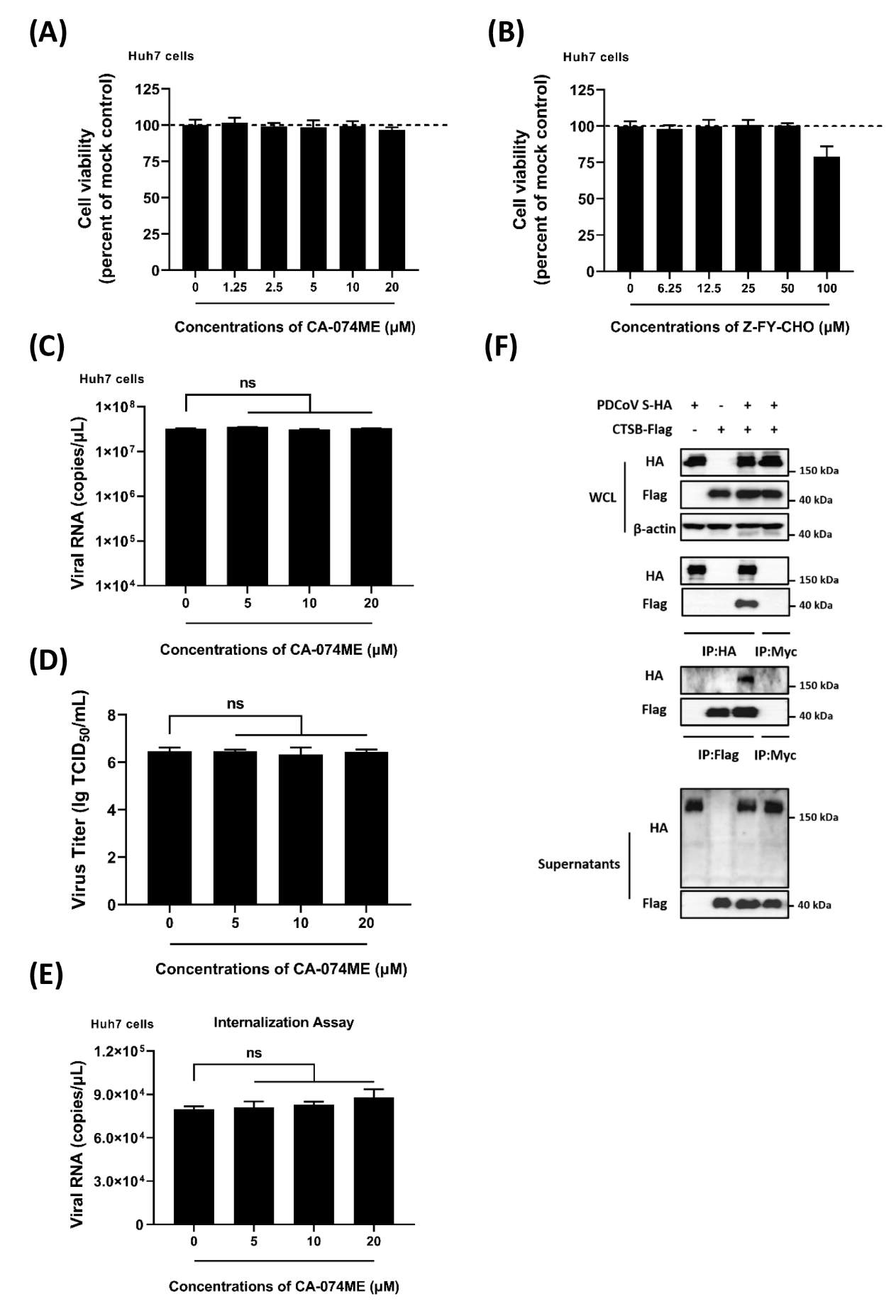
Fig. S4**

**Fig. S4. Endosomal protease cathepsin B does not facilitates PDCoV infection in Huh7 cells. (A, B)** The cytotoxicity of CA-074ME and Z-FY-CHO. Huh7 cells were treated with 0-20 μM of CA-074ME (A) or 0-100 μM of Z-FY-CHO (B) for 24 h, followed by a CCK-8-based cell viability assay in accordance with the manufacturer’s protocols. **(C, D)** Effect of CA-074ME on PDCoV infection. Huh7 cells were pretreated with different concentrations of CA-074ME (0, 5, 10, 20 μM), then the cells were infected with PDCoV^T-^ (MOI=0.5). At 24 hpi, the viral RNA copies and titers were measured by RT-qPCR (C) and TCID_50_ (D) assays, respectively. **(E)** Viral internalization assay with CA-074ME. Huh7 cells were pretreated with different concentrations of CA-074ME (0, 5, 10, 20 μM), then the viral internalization assay was performed with PDCoV^T-^ (MOI=1) and the internalized viral RNA copies were quantified by RT-qPCR. **(F)** Interaction and cleavage between PDCoV S and CTSB. PDCoV S and CTSB were co-transfected in HEK-293T cells, and Co-IPs were performed with indicated antibodies. The anti-Myc tag antibodies served as negative controls. The supernatants from the cells co-expressing PDCoV S and CTSB were collected and then added with precooled acetone overnight at -20°C. After centrifugation, the resulting pellets were air-dried to remove residual acetone, and then resuspended in RIPA lysis buffer for western blot analysis.

**
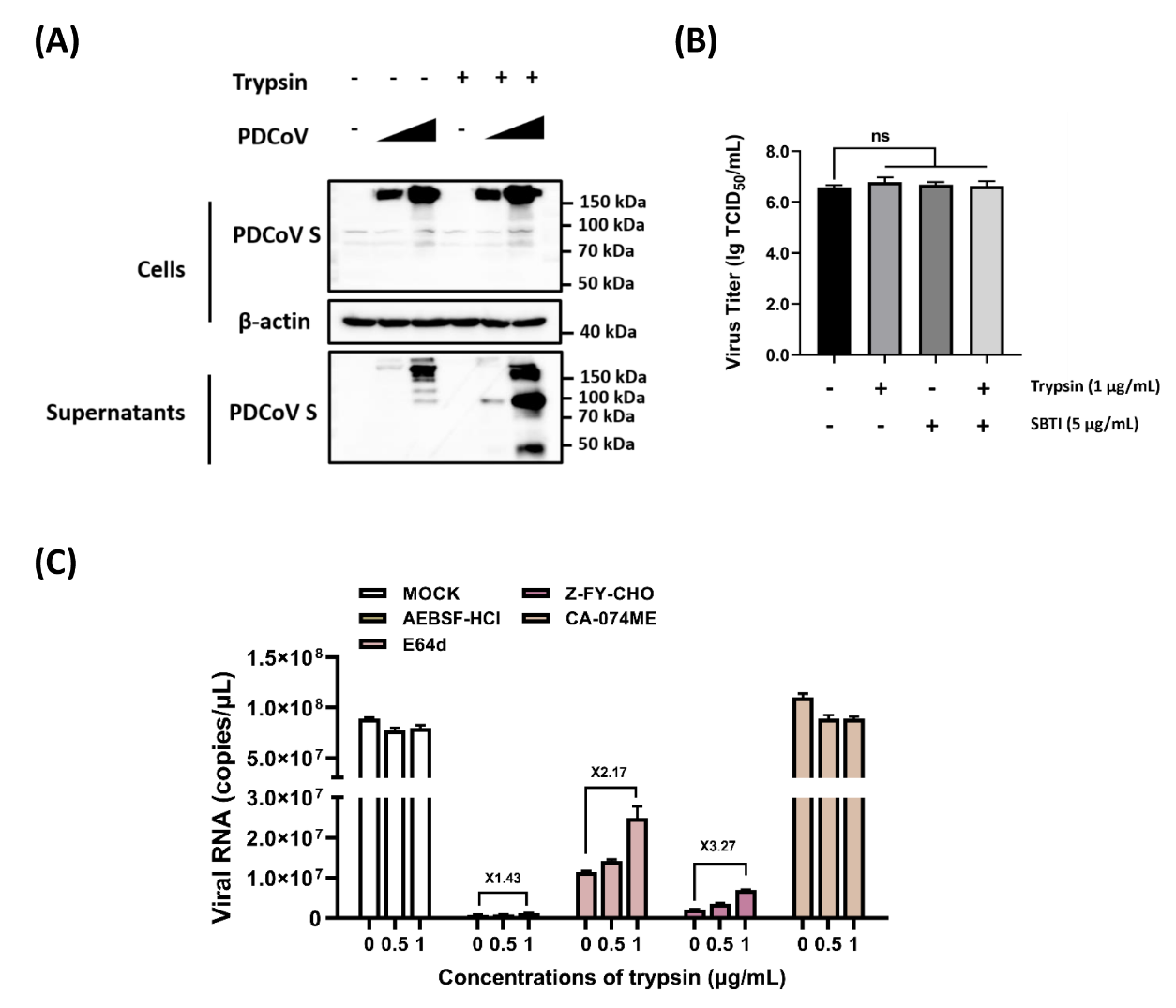
Fig. S5**

**Fig. S5. PDCoV infection in Huh7 cells is independent of trypsin activity. (A)** The cleavage of PDCoV S protein by trypsin. Huh7 cells were infected with different dose PDCoV^T-^ with trypsin or not. At 24 hpi, the cells and supernatants were collected and detected by western blot analysis. **(B)** PDCoV^T-^ (MOI=0.5) was inoculated on Huh7 cells with or without trypsin (1 μg/mL). SBTI (5 μg/mL) was added to quench trypsin activity. At 24 hpi, cells were harvested and viral titers were measured by TCID_50_ assay. (C) Huh7 cells were pretreated with E64d, AEBSF-HCl, CA-074ME, or Z-FY-CHO at 37°C for 1 h, subsequently, different concentrations of trypsin were added during PDCoV^T-^ infection. At 24 hpi, cells were harvested and the viral RNA copies were measured by RT-qPCR.
